# Suppressing Photothermal Artifacts in Flexible Fluorescent Fiber‐Optic Sensors for Accurate and Precise In Situ Temperature Monitoring

**DOI:** 10.1002/advs.78130

**Published:** 2026-10-04

**Authors:** Sijie Su, Jinjian Yu, Pengyuan Dong, Xin Wang, Jing Lyu, Jiulin Gan, Enhai Song, Qinyuan Zhang

**Affiliations:** ^1^ State Key Laboratory of Luminescent Materials and Devices Guangdong Provincial Key Laboratory of Fiber Laser Materials and Applied Techniques South China University of Technology Guangzhou China; ^2^ Guangdong Energy Group Science and Technology Research Institute Co., Ltd. Guangzhou China; ^3^ School of Materials and Engineering Guangdong Engineering Technology Research and Development Center of Special Optical Fiber Materials and Devices South China University of Technology Guangzhou China

**Keywords:** fiber‐optic sensor, fluorescent thermometry, in situ detection, photothermal effect, precision temperature sensing

## Abstract

Ratiometric flexible fluorescent fiber‐optic sensors, featuring self‐calibration and precise spatial localization, are emerging as a robust platform for in situ thermal monitoring, advancing applications such as battery safety management and biomedical diagnostics. However, measurement artifacts induced by the inherent photothermal effect remain largely overlooked, posing a challenge to high‐accuracy temperature sensing. Here, we propose a partial‐inner‐cladding optical fiber (PICOF) to suppress photothermal artifacts by isolating the sensing layer from the coupling interface and the fiber core, where self‐heating predominantly occurs. Compared with conventional fiber structures, the PICOF extends the allowable excitation power from ≤ 15 to 61 mW and improves the luminescence signal‐to‐noise ratio from 36.6 to 42 dB, enabling high‐fidelity readout with a relative standard deviation below 0.09% even under power fluctuations and deformation disturbances. Consequently, the LuVO_4_:25%Yb^3+^/Er^3+^‐composite PICOF achieves record‐high accuracy (maximum absolute error *δ*_max_ = 0.104°C), precision (standard deviation *σ* = 0.05°C), and temperature resolution (*T*_R_ = 0.2°C) among reported fluorescent fiber‐optic sensors. Furthermore, practical demonstrations underscore its utility for early warning of thermal hazards and incipient failure in sodium‐ion batteries, and as an in situ diagnostic reference for the Bárány caloric test. This work provides an effective and generalizable strategy for the advancement of high‐accuracy fluorescent thermometry.

## Graphical abstract

A partial‐inner‐cladding optical fiber (PICOF) is developed to suppress photothermal artifacts in fluorescent fiber‐optic thermometry by isolating the sensing layer from regions prone to photothermal effects. This structural design strategy enables high‐fidelity operation at elevated excitation power, achieving high accuracy (maximum absolute error *δ*_max_ = 0.104°C), precision (standard deviation *σ* = 0.05°C), and resolution (0.2°C) in the LuVO_4_:Yb^3+^/Er^3+^‐composite PICOF.

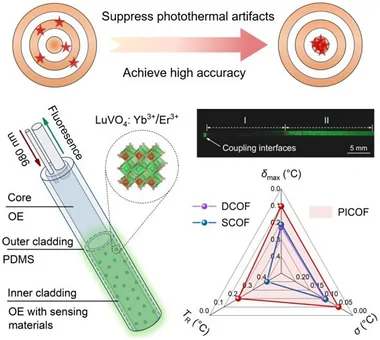

## 1 Introduction

Accurate and precise temperature measurement is essential for deciphering the underlying mechanisms of physical, chemical, and physiological processes, particularly in complex and enclosed systems [1]. Such applications demand sensors capable of suppressing artifacts arising from noise interference to enable reliable monitoring of both static temperature and dynamic thermal variations. To date, resistance‐type sensors have made notable progress in wearable devices [2, 3] and safety monitoring systems [4, 5], owing to their high sensitivity and high integration. In addition, infrared thermography has also been widely adopted owing to its convenient remote thermal imaging capability. However, both of them suffer from intrinsic susceptibility to environmental noise [6, 7] (e.g., electromagnetic interference for electronic components or optical blockage loss for free‐space light), often compromising reliability in precision temperature measurement. In contrast, fiber‐optic sensors combine low transmission loss, inherent immunity to electromagnetic interference, fast response, compact size, and excellent corrosion resistance, enabling low‐noise, real‐time, in situ temperature monitoring in complex environments [8, 9, 10]. Among them, ratiometric fluorescent fiber‐optic sensors integrate flexible waveguides with dual‐emission thermosensitive materials to realize self‐calibrated readout via the fluorescence intensity ratio (FIR). This technique relies on the thermally coupled levels (TCLs) of luminescent centers, whose population distribution follows the Boltzmann distribution law [11]. Such behavior enables the FIR to depend exclusively on temperature, thereby offering strong anti‐interference capability against external crosstalk such as stress, mechanical deformation, and signal intensity fluctuations [11, 12]. To date, significant progress has been achieved in developing such sensors based on various thermosensitive phosphors (e.g., NaYF_4_:Yb^3+^/Er^3+^ [12, 13, 14] and NaLaTi_2_O_6_:Yb^3+^/Er^3+^ [15]) and fiber structures (e.g., core‐cladding structure [12] and dual‐cladding structure [16]), enabling applications in complex systems including rechargeable batteries [15, 17, 18, 19] and the human body [12, 13, 14, 16]. Recently, efforts have focused on enhancing luminescence intensity to improve signal stability [20] or engineering energy‐level structures to boost temperature sensitivity [21], thereby mitigating the impacts of background noise. However, despite these advances in fluorescent fiber‐optic sensors, the achievable accuracy (typically above 0.12°C) and resolution (typically above 0.5°C) in representative reports remain limited. Moreover, measurement distortion can still arise under operating power fluctuations [22]. These observations highlight the presence of overlooked intrinsic noise that fundamentally constrains sensor performance, indicating an urgent need to uncover and suppress such artifacts for more accurate temperature sensing.

Notably, FIR‐based thermometry generally assumes that the temperature of the sensing material faithfully reflects the ambient temperature. However, this assumption can break down due to the photothermal effect induced by non‐radiative processes during photoluminescence. This effect leads to excitation‐power‐dependent self‐heating [23], which inevitably introduces a temperature offset between the sensing material and its surroundings, thereby producing thermal artifacts that mask the true ambient temperature signals. Under realistic conditions involving excitation power fluctuations, such artifacts act as an inherent noise source that fundamentally degrades thermometric fidelity. Reducing excitation power or employing pulsed modulation can mitigate this effect [23], but the accompanying reduction in luminescence signal‐to‐noise ratio (SNR) also impairs temperature measurement performance. Recently, post‐processing methods have been proposed to calibrate the influence of excitation power while maintaining signal intensity [24, 25]. However, these complex demodulation schemes rely on a known excitation power, rendering them impractical for realistic scenarios where incident power on the sensing material is unknown and dynamically fluctuating. More recently, phonon‐assisted energy transfer has been demonstrated to mitigate self‐heating in phosphors by converting phonons into photons [18]. However, this approach lacks general applicability across the diverse range of photoluminescent systems. Moreover, even with this promising strategy, achieving a temperature resolution below 0.5°C when integrated into fiber sensors remains challenging. Therefore, developing an effective and general strategy to suppress photothermal artifacts remains a significant challenge, and a systematic understanding of photothermal effects in fiber‐optic thermometry remains an urgent need.

In this work, the dominant origins of photothermal artifacts in fluorescent fiber‐optic temperature sensors were investigated, identifying the coupling interface and the fiber core, where excitation energy is concentrated, as the regions prone to self‐heating. Based on this insight, we propose a novel partial‐inner‐cladding optical fiber (PICOF) that isolates the sensing layer from these self‐heating regions, effectively suppressing photothermal artifacts through spatial decoupling. A ratiometric thermosensitive phosphor, LuVO_4_:25%Yb^3+^/3%Er^3+^ (LVOYE), was integrated into the fiber to validate this design. Compared with conventional fiber sensors featuring core‐cladding or double‐cladding structures, the PICOF extends the allowable excitation power from ≤ 15 to 61 mW, enhancing anti‐interference capacity against photothermal noise and improving the luminescence SNR from 36.6 to 42 dB. Benefiting from these, the LVOYE‐composite PICOF achieves record‐high temperature measurement accuracy (maximum absolute error *δ*_max_ = 0.104°C), precision (standard deviation *σ* = 0.05°C), and temperature resolution (*T*_R_ = 0.2°C), while maintaining fidelity even under power fluctuations and deformation disturbances. Furthermore, practical demonstrations underscore the PICOF's feasibility for in situ precision temperature monitoring, decoding subtle temperature variations in a sodium‐ion battery for early warning of battery degradation and thermal risks, and providing a promising temperature reference in the ear canal during the Bárány caloric test for precise vestibular diagnosis. This work not only develops a reliable sensing device for in situ precision temperature measurement but also offers an effective and generalizable strategy for developing high‐accuracy fluorescent fiber‐optic temperature sensors.

## 2 Results and Discussions

### 2.1 Design of the PICOF Temperature Sensor

Figure 1a illustrates the upconversion mechanism of the Yb^3^^+^/Er^3^^+^ co‐doped fluorescent material, with the inset detailing the temperature‐response mechanism based on the thermally coupled levels (^2^H_11/2_ and ^4^S_3/2_) of Er^3+^. The thermal population between TCLs remains stable at a fixed temperature, yet rapidly re‐equilibrates according to the Boltzmann distribution upon temperature change [11]. This results in a FIR that depends exclusively on temperature, enabling self‐calibrated temperature monitoring, as described by [1]:

(1) $$\mathrm{FIR}=\frac{I_{H}}{I_{S}}=A\exp\left(\frac{-\Delta E}{k_{B}T}\right)$$

where *I*_H_ and *I*_s_ are the emission intensities of the ^2^H_11/2_ → ^4^I_15/2_ and ^4^S_3/2_ → ^4^I_15/2_ transitions, respectively, and *T* is the temperature of the fluorescent material, which is generally taken to represent the ambient temperature being measured. However, the photothermal effect can induce self‐heating in the material, resulting in a discrepancy between *T* and the true ambient temperature. To mitigate the impact of this effect on sensing performance and enhance sensing fidelity, this work begins by systematically investigating the origin of the photothermal effect in both the sensing material and the optical fiber.

**FIGURE 1:**
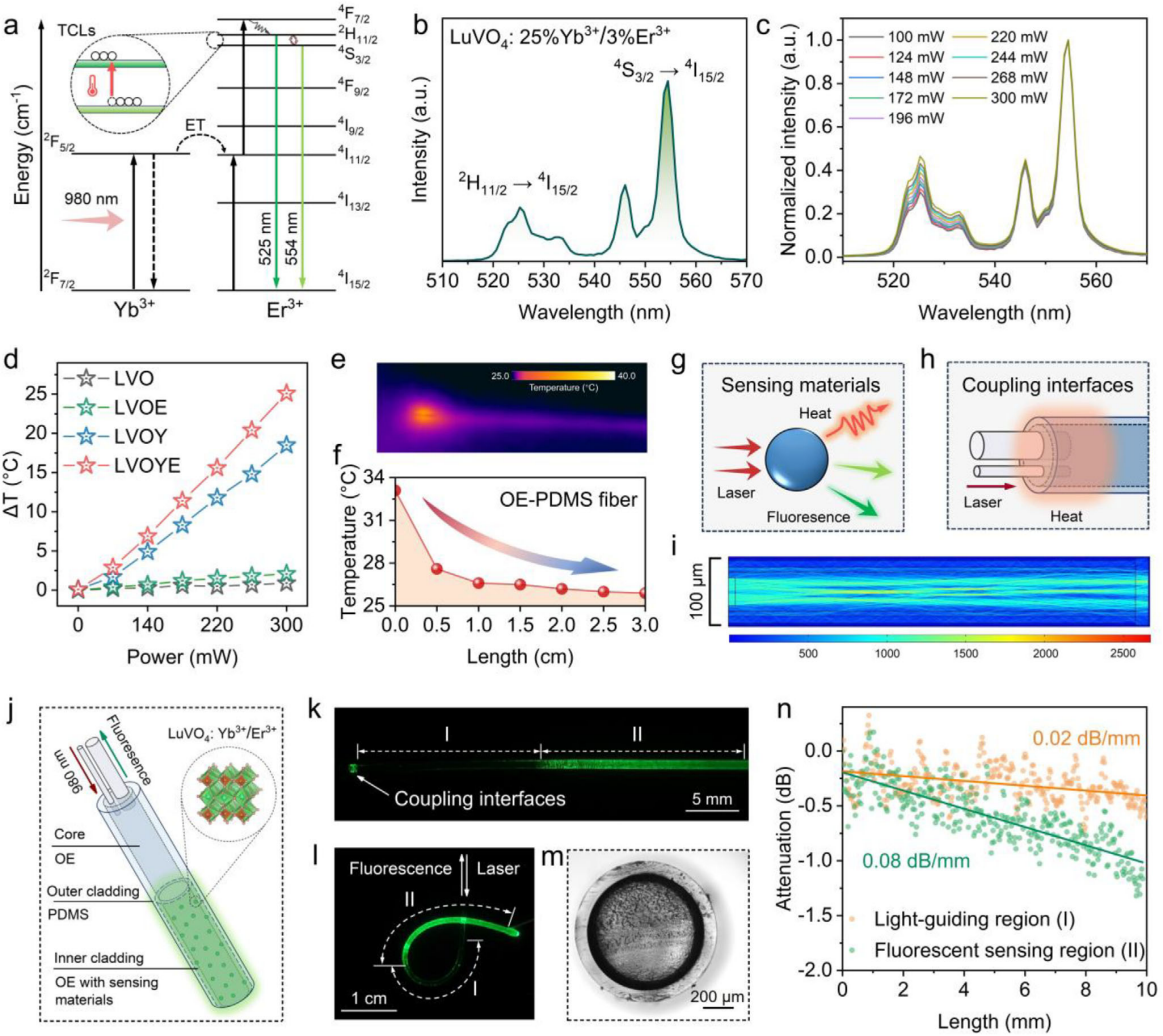
Analysis, design, and structure of the partial‐inner‐cladding fluorescent fiber‐optic sensor to suppress photothermal artifacts. (a) Schematic diagram of the upconversion process in LuVO_4_:Yb^3+^/Er^3+^. The illustration shows the thermal population between thermally coupled levels. (b) Upconversion emission spectrum of LuVO_4_:25%Yb^3+^/3%Er^3+^ under 980 nm laser excitation at 100 mW. (c) Excitation‐power‐dependent upconversion emission spectra of LuVO_4_:25%Yb^3+^/3%Er^3+^ at room temperature. (d) Surface temperature variations of LuVO_4_ (LVO), LuVO_4_:25%Yb^3+^ (LVOY), LuVO_4_:3%Er^3+^ (LVOE) and LuVO_4_:25%Yb^3+^/3%Er^3+^ (LVOYE) samples at various excitation powers. The temperature rise (Δ*T*) is defined as Δ*T* = *T*_max_ – *T*_0_, where *T*_max_ is the maximum surface temperature of the phosphor and *T*_0_ is the ambient temperature. (e, f) Axial temperature distribution in the optical fiber coupled with a 980 nm laser at 200 mW. (g, h) Schematic diagrams of the photothermal effect in the optical fiber (g) and the phosphor (h). (i) Finite element simulation of the optical field distribution in an OE‐OE‐PDMS double‐cladding fiber. (j–m) Structural schematic (j), photographs (k, l), and cross‐sectional microscope image (m) of a partial‐inner‐cladding optical fiber (PICOF) integrated with LuVO:25%Yb^3+^/3%Er^3+^. Regions I and II represent the light‐guiding region without fluorescent material and the sensing region integrated with fluorescent material, respectively. (n) Optical attenuation as a function of the length of the PICOF.

A series of phase‐pure LuVO_4_:Yb^3^^+^/Er^3^^+^ phosphors were synthesized via solid‐state reaction. Among these, LuVO_4_:25%Yb^3^^+^/3%Er^3^^+^ was identified as the optimal composition, delivering the highest upconversion emission intensity and thus a sufficient luminescence SNR, as shown in Figures S1 and S2. The Rietveld refinement results for the LVOYE sample are provided in Figure S3, further confirming its phase purity and crystal structure. Under 980 nm laser excitation, LVOYE exhibits two distinct emission bands at 515–540 nm and 540–565 nm (Figure 1b), corresponding to the ^2^H_11/2_ → ^4^I_15/2_ and ^4^S_3/2_ → ^4^I_15/2_ transitions of Er^3+^, respectively. The normalized excitation‐power‐dependent emission spectra of LVOYE were acquired at room temperature, as shown in Figure 1c. Notably, as the excitation power increases progressively, the intensity of the ^2^H_11/2_ → ^4^I_15/2_ transition shows a relative enhancement compared with that of the ^4^S_3/2_ → ^4^I_15/2_ transition. This observation deviates from the established principle that the FIR from TCLs should be independent of excitation power. We attribute this phenomenon to the photothermal effect within the photoluminescent systems. To verify this, thermal imaging was performed on a series of LuVO_4_ samples under 980 nm excitation at various powers (Figure 1d and Figures S4–S7). The results confirm a clear rise in the phosphor surface temperature with increasing power, directly linking the anomalous power‑dependent FIR to the photothermal effect in Yb^3^^+^/Er^3^^+^ co‑doped systems. In addition, the photothermal effect diminished sequentially across LuVO_4_:25%Yb^3+^/3%Er^3+^, LuVO_4_:25%Yb^3+^, and LuVO_4_:3%Er^3+^ phosphors, while the surface temperature of the undoped LuVO_4_ matrix remained virtually constant. These findings reveal that the photothermal effect originates from both the Yb^3+^ sensitizer and Er^3+^ activator, yet is dominated by Yb^3+^ owing to its large absorption cross‐section at 980 nm [26, 27], combined with the typically low upconversion quantum yield (UCQY) in Yb^3^^+^/Er^3^^+^ codoped systems [28]. In LuVO_4_:25%Yb^3+^/3%Er^3+^, the UCQY is measured to be 0.101% (Figure S8), demonstrating that most of the absorbed photon energy is dissipated as heat through non‐radiative pathways, including multi‐phonon relaxation within Er^3+^ (e.g., ^4^F_7/2_ → ^2^H_11/2_/^4^S_3/2_ and ^4^I_11/2_ → ^4^I_13/2_) [29] and energy migration to quenching centers [30]. Furthermore, the photothermal effect in the polymer optical fiber (POF) is also non‐negligible, as shown in Figure 1e,f. Upon coupling a 980 nm laser into the POF through a tail fiber, the fiber temperature reached the maximum value near the coupling interfaces owing to the pronounced optical loss, then decreased gradually along the propagation direction. Taken together, the photothermal effect in the fluorescent fiber‐optic sensor originates from two primary sources: (i) non‑radiative energy conversion in the sensing material (Figure 1g), and (ii) pronounced optical loss near the coupling interfaces between the sensing fiber and the tail fiber (Figure 1h). In addition, it is worth noting that excessive excitation power density in the fiber core (Figure 1i) may exacerbate the non‐radiative energy conversion and thereby cause significant heating.

To overcome the degradation in robustness and accuracy of fiber‐optic temperature sensors induced by the photothermal effect, we designed a novel partial‐inner‐cladding optical fiber (PICOF) structure based on the foregoing analysis, as shown in Figure 1j. The PICOF sensor comprises an optical encapsulant (OE) core and a polydimethylsiloxane (PDMS) cladding for light guidance, a phosphor‐composite partial inner cladding for temperature response, and quartz pigtails for excitation light delivery and emission fluorescence collection. Differing from the conventional designs of fluorescent composite fiber‐optic sensors, the PICOF exhibits two distinctive features: (i) the fiber is strategically partitioned into a light‐guiding region without sensing material (region I) and a fluorescent sensing region integrated with sensing material (region II). This spatial separation not only effectively isolates the sensing region from interference from the significant photothermal effect around the coupling interfaces but also enables a fully flexible sense‐and‐transmit fiber system to achieve more precise positioning. (ii) The sensing material is incorporated into the inner cladding to mitigate the severe photothermal effect on the phosphor induced by the excessive optical power density in the fiber core, rather than merely to reduce transmission loss. Figure 1k,l present the photographs of the fabricated PICOF, showing its structurally distinct light‐guiding region (I) and fluorescent sensing region (II). The fiber exhibits excellent flexibility and maintains outstanding light‐guiding performance even under bending. A cross‐sectional microscope image (Figure 1m) confirms a concentric and homogeneous morphology along the fiber axis, with a uniform diameter of 900 µm. Propagation loss of the fabricated PICOF was measured by a dark‐field scattering method [31] (Figure 1n). Both the light‐guiding and fluorescent sensing regions exhibit low propagation losses, with coefficients of 0.02 and 0.08 dB/mm, respectively.

### 2.2 Performance of the PICOF in Suppressing Photothermal Artifacts

As illustrated in Figure 2a and detailed in the Supporting Information, LVOYE‐composite fluorescent fiber‐optic sensors featuring core‐cladding (CC), double‐cladding (DC), segmented‐core (SC), and partial‐inner‐cladding (PIC) structures were fabricated using a mold method with spin coating. These fibers, which represent a progressive evolution with the sensing layer gradually isolated from the coupling interface and fiber core, were designed to investigate the specific influence of the photothermal effect and to compare the photothermal interference suppression performance among different structures. Upon excitation by a 980 nm laser coupled through a tail fiber, all sensors exhibited bright green emission from the LVOYE phosphor in the sensing regions, but displayed distinct light‐guiding properties (Figure 2b). The emitted fluorescence was collected by another tail fiber and recorded with a spectrometer (Figure S9), enabling facile monitoring of the FIR corresponding to the ^2^H_11/2_/^4^S_3/2_→ ^4^I_15/2_ transitions of Er^3+^.

**FIGURE 2:**
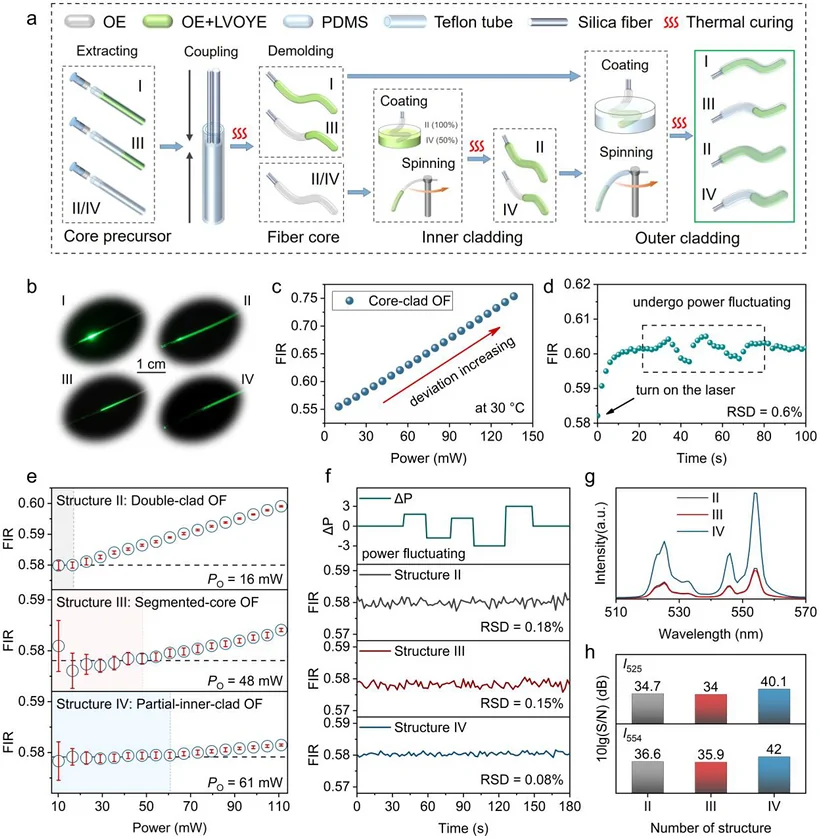
Comparative analysis of the photothermal interference suppression performance in LVOYE‐composite fiber‐optic sensors with different structures. (a, b) Fabrication schematic (a) and photographs (b) of the (I) core‐cladding (CC), (II) double‐cladding (DC), (III) segmented‐core (SC), and (IV) partial‐inner‐cladding (PIC) fiber‐optic sensors. (c) Excitation‐power‐dependent FIR of the CC fiber‐optic sensor; (d) Continuous‐output FIR from the CC fiber‐optic sensor under excitation power fluctuation at 30°C. (e) Excitation‐power‐dependent FIR of the DC, SC, and PIC fiber‐optic sensors. *P*_O_ is defined as the optimal operating power. (f) Continuous‐output FIR from the DC, SC, and PIC fiber‐optic sensors operating at *P*_O_ under 30°C with power fluctuations. The power deviation (Δ*P*) is defined as Δ*P = P*_in_ ‐*P*_O_, where *P*_in_ denotes the input excitation power. (g, h) Upconversion spectra (g) and the corresponding SNR (h) of the DC, SC, and PIC fiber‐optic sensors operating at *P*_O_.

Notably, since the FIR depends exclusively on the temperature of the sensing material, the photothermal effect can be self‑monitored by measuring the FIR under varying excitation powers at a constant ambient temperature. Figure 2c shows the excitation‐power‐dependent FIR of the CC fiber‐optic sensor at 30°C, which exhibits a monotonic increase from 10 to 136 mW, indicating a progressively stronger photothermal effect and larger measurement deviation. Furthermore, a modulated input power (Figure S10) was introduced to the CC fiber sensor to simulate the power fluctuations in practical applications. The corresponding FIR response (Figure 2d) shows an initial rapid rise upon laser turn‑on, followed by stabilization as the photothermal effect saturates at a fixed power. During the subsequent power modulation, the FIR tracked the input power, increasing and decreasing accordingly. The relative standard deviation (*RSD*) was employed to quantify measurement fluctuations, as calculated by [32]:

(2) $$\mathrm{RSD}\;=\sqrt{\frac{\sum_{j=1}^{n}\left(\mathrm{FI}R_{j\;}-\;\bar{\mathrm{FIR}}\right)}{n-1}}\times \frac{1}{\bar{\mathrm{FIR}}}\times 100\%\;$$

where *FIR_j_* is the individual measured *FIR*, $\bar{FIR}$ is the mean value over multiple measurements, and *n* is the number of measurements. The *RSD* of the CC fiber‐optic sensor under power fluctuations was calculated to be 0.6%, demonstrating how photothermal interference degrades practical measurement accuracy and precision.

The optimal operating power (*P*_O_) for fluorescent temperature sensors was proposed in this work to quantitatively evaluate the photothermal interference suppression performance and standardize the conditions of operational power. *P*_O_ is defined as the maximum allowable excitation power for stable readout, specifically the power threshold beyond which the FIR no longer remains stable but instead increases with rising excitation power. As shown in Figure 2e, the excitation‐power‐dependent FIR of the DC, SC, and PIC fiber‐optic sensors was measured at a constant temperature of 30°C. The results reveal that each as‐fabricated fiber exhibits a well‐defined *P*_O_ of 16 mW for DC fiber, 48 mW for SC fiber, and 61 mW for PICOF. The higher *P*_O_ of the PICOF underscores its superior robustness against photothermal interference, benefiting from the spatial decoupling strategy. This robustness was maintained even under excitation power fluctuations, with an RSD value of 0.08% for PICOF (Figure 2f). Notably, the variation in *P*_O_ suggests that reducing excitation power could mitigate photothermal interference across different fiber structures. However, the concomitant reduction in emission intensity degrades the SNR, which in turn impairs both the precision and accuracy of temperature measurement. The luminescence SNR of LVOYE‐composite fiber sensors was calculated from Figure 2g using the following equation [33]:

(3) $$SNR=10\mathrm{lo}g_{10}\left(\frac{P_{S}}{P_{N}}\right)$$

where *P*_S_ and *P*_N_ represent the average optical powers calculated from the integrated intensity of the emission peak and the background noise, respectively. As shown in Figure 2h, the PICOF exhibits an SNR of 42 dB at *P*_O_, surpassing both the CC (36.6 dB) and the SC (35.9 dB) fiber‐optic sensors. Consequently, the PICOF combines an excellent luminescence SNR with outstanding photothermal interference suppression performance, demonstrating strong potential for highly accurate and precise temperature measurements.

### 2.3 Temperature Sensing Performance of the PICOF Sensor

The as‐fabricated LVOYE‐composite PICOF sensor was placed in a temperature controller with a precision of 0.01°C to evaluate its temperature sensing performance. All characterizations were performed at the optimal operating power (*P*_O_ = 61 mW) of the PICOF sensor. Figure 3a shows the normalized upconversion emission spectra at different temperatures. The FIR of emission peaks at 525 and 554 nm (*I*_525_/*I*_554_) exhibited a strong Boltzmann‐type temperature dependence, arising from the thermal coupling between ^2^H_11/2_ and ^4^S_3/2_ levels in Er^3+^. Owing to the proven photothermal interference suppression performance of PICOF, the FIR demonstrates exclusive sensitivity to the ambient temperature, rendering it an ideal characteristic parameter for temperature sensing. Derived from Equation 1, ln(*I*_525_/*I*_554_) versus 1/*T* can be linearly fitted as:

(4) $$\ln\left(\frac{I_{525}}{I_{554}}\right)=\frac{-\Delta E}{k_{B}}\times \frac{1}{T}+C$$

where *C* is the fitting factor, *k*_B_ is the Boltzmann constant, Δ*E* is the energy gap between ^2^H_11/2_ and ^4^S_3/2_, and *T* is the ambient temperature to be measured. As shown in Figure 3b, the values of Δ*E* and *C* were determined to be 776.7 cm^−1^ and 3.14, respectively, with a high coefficient of determination (*R* ^2^ = 0.9999). Because the FIR values are consistent at the same temperature during both heating and cooling cycles without hysteresis (Figure 3c), Equation 4 is universally applicable to both processes. These results indicate that the target temperature can be readily obtained by solving the equation. The response speed of the sensor was then evaluated by comparison with a commercial thermistor (Figure 3d). The response curves of the PICOF sensor can almost synchronize with the thermistor during both heating and cooling processes, validating its rapid tracking capability to continuous temperature variations. In addition, its step response was assessed by monitoring the readouts upon immersion from air into warm water, indicating a short response time of 2 s (Figure S11).

**FIGURE 3:**
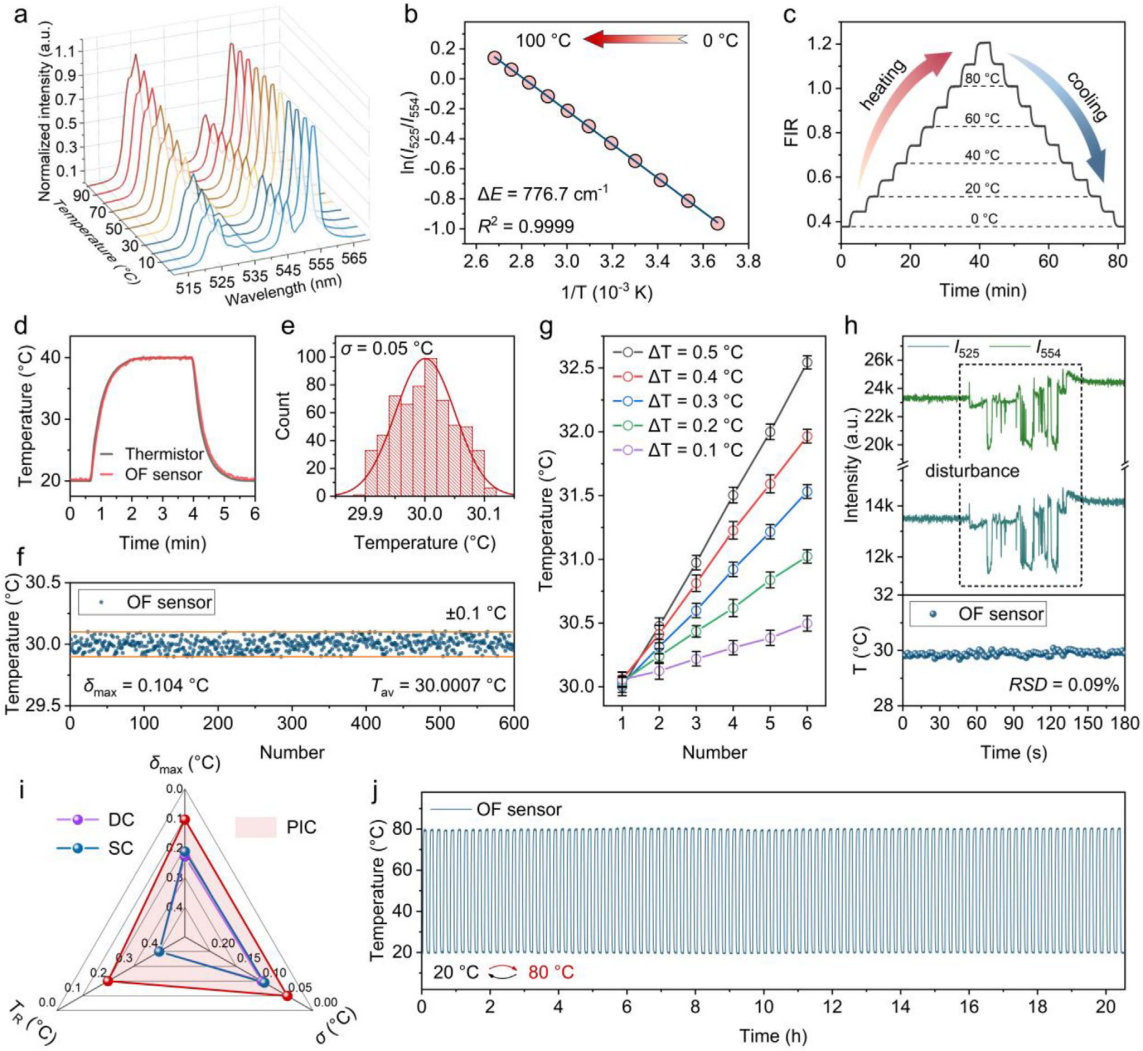
Temperature sensing performance of the LVOYE‐composite PICOF sensor. (a) Temperature‐dependent emission spectra of the PICOF sensor under 980 nm excitation. (b) Linear fitting plot of ln(*I*_525_/*I*_554_) versus 1/*T*. *I*_525_ and *I*_554_ were calculated based on the integrated intensity of emission peaks centered at 525 and 554 nm, respectively. (c) Temperature‐cycling test between 0°C and 100°C at an interval of 10°C. (d) Dynamic temperature response of the PICOF sensor compared with a commercial thermistor. (e) Distribution and Gaussian fitting results of the measured temperature at 30°C from the PICOF sensor. (f) The continuous monitoring results and measurement deviation from the PICOF sensor at 30°C. (g) Practical temperature resolution of the PICOF sensor. (h) Continuous monitoring results of *I*_525_, *I*_554_, and the measured temperature of PICOF calculated based on *I*_525_/*I*_554_ values under various mechanical interferences, including bending, swinging, and stretching. (i) Comparison of accuracy (*δ*_max_), precision (*σ*), and temperature resolution (*T*_R_) among the DC, SC, and PIC fiber‐optic sensors. (j) 100‐cycle test of the PICOF sensor with the ambient temperature periodically changing between 20°C and 80°C.

The accuracy and precision of the sensor were quantitatively evaluated by its maximum absolute error (*δ*_max_) and standard deviation (*σ*), respectively. For characterization, the fiber‐optic sensor was subjected to continuous temperature monitoring at a constant temperature of 30°C. Gaussian distribution analysis of the data revealed that 99% of the measured values fell within the range of ± 0.10°C, with a standard deviation of 0.05°C, confirming high precision (Figure 3e). Additionally, over the course of 600 measurements, the *δ*_max_ between the measured and set temperature was only 0.104°C, and the average value of multiple measurements was 30.0007°C, with only a 0.0007°C deviation from the set value, indicating exceptional accuracy of the as‐fabricated temperature sensor (Figure 3f). The practical temperature resolution (*T*_R_), defined as the minimum detectable temperature change, was evaluated by applying successive temperature steps (Figure 3g). The high precision, accuracy, and sensitivity (Figure S12) of the PICOF sensor enabled an outstanding *T*_R_ of 0.2°C, demonstrating its capability to distinguish minute temperature variations. Moreover, to evaluate the robustness of the PICOF sensor against mechanical deformation in practical applications, it was subjected to disturbances induced by stretching, swinging and bending. As shown in Figure 3h, although *I*_525_ and *I*_554_ exhibited pronounced fluctuations, the corresponding temperature readings maintained exceptional stability, with a low *RSD* of 0.09%. For comparison, the temperature sensing performance of the CC and SC fiber‐optic sensors was also characterized at their corresponding *P*_O_, validating that the PICOF structure with excellent photothermal interference suppression performance significantly enhances the accuracy, precision, and resolution of fluorescent temperature sensors (Figure 3i and Figures S13 and S14). Additionally, it is worth noting that the as‐fabricated LVOYE‐composite PICOF sensor ranks among the best reported for the combined accuracy, precision, and resolution among the reported fluorescent composite optical fiber sensors [12, 13, 14, 15, 16, 17, 18, 20, 22, 32, 34, 35, 36, 37, 38] (Table 1). The repeatability and operational durability were further assessed through consecutive heating–cooling cycling tests (Figure 3j). The sensor exhibited highly reproducible responses over 100 cycles, confirming its excellent stability and repeatability for long‐term operation.

**TABLE 1:** Accuracy (*δ*_max_), precision (*σ*) and practical resolution (*T*_R_) of reported FIR‐based fiber‐optic temperature sensors.

Sensing material	Structure	Accuracy (*δ*_max_)	Precision (*σ*)	Resolution (*T*_R_)	Ref.
NaYF_4_: Er^3+^/Yb^3+^	core‐cladding OF	—	0.3°C (at 30°C)	—	[12]
NaYF_4_: Er^3+^/Yb^3+^	core‐cladding OF	—	0.19°C (at 37°C)	—	[13]
NaYF_4_: Er^3+^/Yb^3+^	core‐cladding OF		0.4°C (at 25°C)		[14]
NaLaTi_2_O_6_: Yb^3+^/Er^3+^	core‐cladding OF	0.12°C (at 26.5°C)	—	—	[15]
Li_2_ZnSiO_4_: Mn^2+^	dual‐cladding OF	—	0.2°C (at 37°C)	—	[16]
Cs_2_NaLuCl_6_: Yb^3+^/Er^3+^	core‐cladding OF	0.13°C (at 30°C)	0.07°C (at 30°C)	—	[17]
Y_2_Mo_4_O_15_: Yb^3+^/Nd^3+^	dual‐cladding OF	0.12°C (at 37°C)	0.08°C (at 37°C)	0.5°C (30°C–40°C)	[18]
Li_2_HfO_3_:Er^3+^/Yb^3+^	dual‐cladding OF	—	0.1°C (at 30°C)	—	[20]
NaLu(WO_4_)_2_: Er^3+^/Yb^3+^	dual‐cladding OF	0.155°C (at 30°C)	0.065°C (at 30°C)	0.5°C (30°C–35°C)	[22]
Na_2_NbOF_5_: Mn^4+^	cover on Y‐type OF	0.26°C (at 100°C)	—	—	[32]
CaZnOS: Nd^3+^/Er^3+^	cover on Y‐type OF	1.199°C (at 37°C)	—	—	[34]
KYF_4_: Er^3+^	core‐cladding OF	—	0.08°C (at 37°C)	0.5°C (30°C–40°C)	[35]
Rb_3_KCdCl_6_: Mn/Sb	core‐cladding OF	0.2°C (at 28.2°C)	—	—	[36]
CsNaNbOF_5_:Mn^4+^	dual‐cladding OF	0.28°C (at 0°C)	—	—	[37]
ZnS: Mn^2+^	core‐cladding OF	—	0.18°C (at 25°C)	—	[38]
LuVO_4_: Yb^3+^/Er^3+^	dual‐cladding OF	0.229°C	0.10°C	0.4°C	This work^a)^
LuVO_4_: Yb^3+^/Er^3+^	segmented‐core OF	0.212°C	0.095°C	0.4°C	
LuVO_4_: Yb^3+^/Er^3+^	partial inner‐cladding OF	0.104°C	0.05°C	0.2°C	

^a)^ In this work, the accuracy and precision were evaluated at 30°C, while the resolution was characterized over the temperature range of 30°C–32.5°C.

### 2.4 In Situ Precision Temperature Monitoring Applications of the PICOF Sensor

The fabricated PICOF sensor integrates validated robustness, high accuracy, precision, and resolution with a compact size, excellent flexibility, and low propagation loss. This exceptional combination of performance makes it well suited for implantable and in situ temperature monitoring to precisely decode the unknown dynamic processes within complex systems, such as next‐generation batteries and the human body (Figure 4a). Given that in situ devices often operate in liquid environments of varying pH, the sensor's stability was further verified by multiple consecutive recordings of the output FIR in different media. As shown in Figure 4b, the readouts exhibit low dispersion in water, hydrochloric acid, sodium hydroxide, and ethanol, with standard deviations below 0.002, demonstrating outstanding moisture tolerance and corrosion resistance of the PICOF sensor. Leveraging its immunity to electromagnetic and mechanical interference, along with the non‐invasive nature [15, 18], the PICOF sensor was implanted near the positive electrode tab in a sodium‐ion pouch cell for operando temperature monitoring. Meanwhile, the SC fiber‐optic sensor was also implanted at an identical location for comparative analysis, as illustrated in Figure S15. Figure 4c shows the real‐time dynamic temperature profiles captured by the two fiber‐optic sensors during 1 C charge/discharge cycles. As observed, the PICOF sensor reveals finer details in temperature variations, which is of great significance for exploring the relationship between subtle thermal changes and electrochemical processes in the battery. Figure 4d presents the battery's internal temperature variations at different current densities (1–3 C) operando monitored by a PICOF sensor, demonstrating that higher charge/discharge rates lead to more pronounced heat accumulation. Notably, benefiting from the high precision and resolution of the PICOF sensor, the different temperature evolution during two consecutive 3 C charging steps can be clearly distinguished, as shown in Figure 4e. Specifically, the first charge step (I) exhibited a gradual temperature rise with a transient plateau, whereas the subsequent step (II) showed a more rapid increase without a plateau, exceeding the prior peak in a shorter time. This in situ discriminative capacity for different heating rates enables the timely identification of operational anomalies in batteries. Furthermore, as the battery underwent subsequent 3 C cycling, the temperature profile presented a gradually decreasing peak amplitude and an absence of distinct inflection points, reflecting progressive battery degradation and eventual failure (Figure 4f). Thus, real‐time decoding of abnormal thermal behaviors via the high‐precision PICOF sensor enables both timely identification of operational thermal risks and assessment of performance degradation thresholds, providing dual warning for thermal hazards and incipient battery failure. In addition, to validate the sensor stability during operando monitoring, swelling tests and extended temperature monitoring were performed. As shown in Figure S16, PDMS as the outer cladding material exhibits a negligible swelling ratio (<1%) during immersion in the electrolyte, whereas OE as the matrix for the fiber core and inner cladding shows a low swelling ratio (< 5%). Notably, owing to the protection of the PDMS cladding, the PICOF sensor also exhibits a very low swelling ratio (< 1.5%), demonstrating its chemical stability in the electrolyte. Furthermore, the sensor maintains stable temperature readings under prolonged battery cycling at 1 C (Figure S17), underscoring its reliability for operando battery temperature monitoring.

**FIGURE 4:**
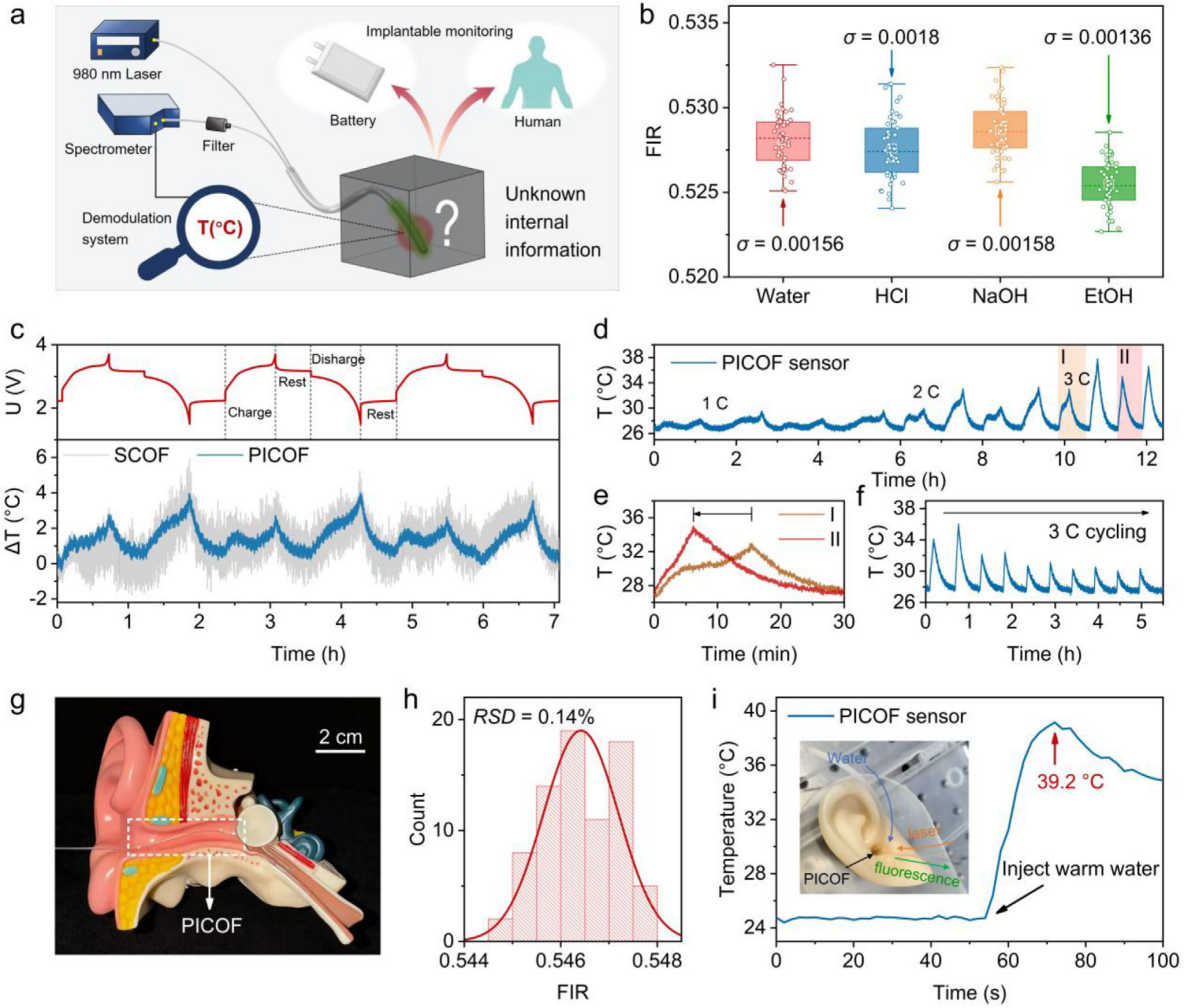
In situ temperature monitoring applications of the LVOYE‐composite PICOF sensor. (a) Schematic diagram of the implantable PICOF sensor for decoding the unknown internal information within a target. (b) Distribution of the FIR readouts from the PICOF sensor in different mediums (water, 0.5 mol/L HCl, 0.5 mol/L NaOH, or ethanol) at room temperature. (c) Charge/discharge voltage profile of a sodium‐ion battery cycled at 1 C, and the operando internal temperature variations during this process simultaneously monitored by segmented‐core optical fiber (SCOF) and PICOF sensors. (d) Operando internal temperature monitoring of the battery at different current densities (1–3 C) at room temperature. (e, f) Internal temperature variations of the battery during the initial (I) and second (II) 3 C charging steps (e), and during the continuous 3 C cycling (f). g) Photograph of the flexible PICOF sensor implanted in a human ear model. (h) Distribution of the FIR readouts from the PICOF sensor in a bionic ear model at room temperature. (i) In situ temperature tracking in a bionic ear model during a simplified Bárány caloric test.

The caloric test, a foundational vestibular diagnostic method for which Robert Bárány was awarded the 1914 Nobel Prize, relies on irrigating a patient's ear canal with warm (≈ 44°C) or cool (≈ 30°C) water to induce nystagmus [39]. However, its clinical accuracy remains constrained by the absence of reliable sensors for in situ monitoring of the actual water temperature in the ear canal or on the tympanic membrane during dynamic irrigation. Leveraging the well‐established biocompatibility of PDMS [16, 40, 41] and the superior thermometric performance and intrinsic flexibility of the PICOF, the sensor holds significant promise for implantable human sensing to address this critical unmet need, where high‑precision temperature monitoring and biocompatibility are both essential. As demonstrated in a human ear model (Figure 4g and Figure S18), the flexible PICOF fully conforms to the winding ear canal without damage, even under substantial deformation. In addition, 100 bending cycles were applied to the fiber to verify its structural integrity after deformation. As shown in Figure S19, cross‐sectional images before and after bending cycles confirm that the fiber retains an intact structure without cladding delamination and sensing material leakage. When implanted in a bionic silicone ear, the sensor's readouts exhibited a low *RSD* of 0.14% at room temperature (Figure 4h), validating its excellent stability in the ear canal. Furthermore, a simplified Bárány caloric test system was constructed using a medical infusion set. Specifically, during the test, warm water at 44°C (measured by an external thermocouple) was poured into the infusion bottle and then delivered into the ear canal through the infusion tubing (inset of Figure 4i). When the warm water was introduced into the ear canal, the PICOF sensor responded rapidly, recording a maximum temperature of 39.2°C (Figure 4i). This result reveals the discrepancy between the temperature inside the ear canal and the externally calibrated water temperature, attributable to heat dissipation during delivery. This real‐time and in situ monitoring capability facilitates dynamic measurement and calibration of irrigation temperature during the Bárány caloric test, thereby exhibiting significant potential to improve the accuracy and reliability of vestibular diagnostics.

## 3 Conclusions

In summary, this work reveals the dominant origins and quantitative impact of the photothermal effect in fluorescent fiber‐optic thermometry, and proposes a novel PICOF structure to suppress this inherent measurement artifact for precision temperature monitoring. The PICOF is designed by isolating the LVOYE‐composite sensing layer from the coupling interface and the fiber core, where self‐heating predominantly occurs due to the concentrated excitation energy. Compared with conventional fiber structures, the PICOF sensor operates free from photothermal artifacts at higher excitation powers (up to 61 mW), and maintains stable readouts even under power fluctuation (*RSD* = 0.08%) and deformation disturbance (*RSD* = 0.09%). Consequently, both high fidelity and luminescence SNR (42 dB) are simultaneously achieved in one fiber sensor, resulting in record‐high accuracy (*δ*_max_ = 0.104°C), precision (*σ* = 0.05°C), and resolution (*T*_R_ = 0.2°C) among the fluorescent fiber‐optic temperature sensors. Additionally, a 100‐cycle test further confirms its stability and repeatability for long‐term operation. Leveraging these advantages, we have successfully constructed a robust sensing system for in situ precise temperature monitoring. This system can timely decode the subtle temperature variations induced by anomalous operational states in sodium‐ion batteries, providing reliable early warning for battery degradation and thermal risks. It also demonstrates feasibility for dynamic temperature measurement during the Bárány caloric test in the ear canal, offering a promising temperature reference to improve the accuracy of vestibular diagnostics. By spatially decoupling sensing from photothermal artifacts, the generalizable PICOF structure represents a significant advancement in the development of high‐accuracy fluorescent fiber‐optic thermometry, extending new application possibilities for emerging precision detection involving minute temperature variations.

## 4 Experimental Section

Detailed experimental procedures and characterizations are reported in the Supporting Information.

## Author Contributions

**Sijie Su**: investigation, writing – original draft, writing – review and editing. **Jinjian Yu**: methodology, investigation, writing – original draft. **Pengyuan Dong**: data curation, methodology, writing – original draft. **Xin Wang**: methodology, formal analysis. **Jing Lyu**: data curation, visualization. **Jiulin Gan**: resources, funding acquisition, writing – review and editing, supervision, conceptualization. **Enhai Song**: investigation, supervision, writing – review and editing, conceptualization. **Qinyuan Zhang**: supervision, conceptualization, writing – review and editing.

## Conflicts of Interest

The authors declare no conflicts of interest.

## Supporting information




**Supporting File**: advs78130‐sup‐0001‐SuppMat.docx.

## Data Availability

The data generated and analyzed during this study are available from the corresponding author upon reasonable request.
