# Supplementary material for: Suppressing Photothermal Artifacts in Flexible Fluorescent Fiber‐Optic Sensors for Accurate and Precise In Situ Temperature Monitoring
Source: Adv Sci (Weinh). 2026 Oct 4:e78130. Online ahead of print. doi: 10.1002/advs.78130 (PMC13634573; doi:10.1002/advs.78130)
Supplement: Supplementary file 1 [file ADVS-9999-e78130-s001.docx]

**Supporting Information**

**Suppressing Photothermal Artifacts in Flexible Fluorescent Fiber-Optic Sensors for Accurate and Precise In Situ Temperature Monitoring**

*Sijie Su, Jinjian Yu, Pengyuan Dong, Xin Wang, Jing Lyu, Jiulin Gan*, Enhai Song* and Qinyuan Zhang**

S. J. Su, J. J. Yu, X. Wang, J. Lyu, Prof. E. H. Song, Prof. Q. Y. Zhang

State Key Laboratory of Luminescent Materials and Devices, Guangdong Provincial Key Laboratory of Fiber Laser Materials and Applied Techniques, South China University of Technology, Guangzhou 510641, China.

Prof. J. L. Gan

School of Materials and Engineering, Guangdong Engineering Technology Research and Development Center of Special Optical Fiber Materials and Devices, South China University of Technology, Guangzhou 510641, China

P. Y. Dong

Guangdong Energy Group Science and Technology Research Institute Co. Ltd, Guangzhou 510000, China

**Experimental Section**

*Materials:* Lutetium oxide (Lu_2_O_3_, 99.99%, metals basis), ytterbium oxide (Yb_2_O_3_, 99.99%, metals basis), erbium oxide (Er_2_O_3_, 99.99%, metals basis) and ammonium vanadate (NH_4_VO_3_, 99.9%, metals basis) were purchased from Aladdin Industrial Corporation. The base and curing agent for forming methylphenyl siloxane (OE 6550) and polydimethylsiloxane (PDMS Sylgard 184) were purchased from Dow Corning Corporation. All raw materials were used without further purification.

*Preparation of LVOYE Phosphor:* LuVO_4_: Yb^3+^/Er^3+^ phosphor samples were synthesized by the solid-state reaction. Firstly, the raw materials were weighted according to the designed stoichiometric ratio and ground thoroughly in an agate mortar. Subsequently, the mixtures were sintered at 900 °C for 1 h followed by regrinding and heating at 1100 °C for 4 h. Finally, the resulting products were collected and reground for further characterization and optical fiber fabrication. All operations were performed in an ambient atmosphere.

*Fabrication of LVOYE-composite POF Sensor:* To form a total internal reflection structure, methylphenyl siloxane (OE 6550, base : curing agent = 1:1, weight ratio) with a high refractive index (*n*_OE_ = 1.514) was selected as the core and inner cladding matrix, polydimethylsiloxane (PDMS Sylgard 184, base : curing agent = 10:1, weight ratio) was selected as the outer cladding matrix. All the optical fibers were fabricated by combining the mold method with the spin-coating technique. For the fiber core fabrication, the OE precursor composited with or without LVOYE phosphor was injected into a Teflon tube mold (inner diameter = 560 μm), and then was cured at 100 °C for 1 h. For the inner cladding fabrication, the core-only fiber was dipped into the OE precursor composited with LVOYE phosphor (OE : phosphor = 40:1, weight ratio), then fixed on the rotating rod, followed by sequential clockwise and counterclockwise spin‑coating at 1000 rpm to ensure uniformity. Subsequently, this coated core was cured at 100 °C for 1 h to consolidate the inner cladding. For the outer cladding fabrication, the fiber was dipped into the PDMS precursor, then fixed on the rotating rod, followed by sequential clockwise and counterclockwise spin‑coating at 1000 rpm to ensure uniformity. After curing at 90 °C for 40 min, the outer cladding was consolidated, and the LVOYE-composite POF was obtained. Two multi-mode silica fibers with diameters of 125 μm and 330 μm were inserted into the end of the fiber core as tailed fibers before core curing.

*Characterization:* The X-ray diffraction (XRD) pattens of LuVO_4_:y%Yb^3+^/x%Er^3+^ for phase analysis were determined by an Aeris powder X-ray diffractometer (PANalytical Corporation, Nether lands) using Cu K𝛼 radiation (𝜆 = 1.5406 Å). The UC emission spectra under 980 nm excitation of LuVO_4_:y%Yb^3+^/x%Er^3+^ and LVOYE-composite flexible fiber-optic sensors were collected by a fiber optic spectrometer (QE pro, Ocean Optics) with a 50-micron slit. During the FIR measurement, the integration time of the spectrometer was set to 500 ms, and the readings were averaged every four measurements. The thermal imaging photographs were obtained by an infrared thermal camera (Fotric 225s). The cross-sectional photograph of the as-fabricated optical fiber was recorded by an optical microscope (Zeiss Imager Z2). The optical power for attenuation calculation was measured by a dark-field scattering method. Temperature calibration and sensing performance were evaluated using a custom-built temperature controller (precision: 0.01 °C) integrated with a commercial thermistor. The pouch-type sodium-ion battery (capacity: 1 Ah) for implantable application demonstration was prepared by the Guangdong Rongna New Energy Technology Co., Ltd., and the charge/discharge tests were carried out on a Neware battery testing system (CT-4008T, Shenzhen, China) at voltage range of 1.5 V -3.7 V. The upconversion quantum yield (UCQY) of LuVO_4_:25%Yb^3+^/3%Er^3+^ was measured by an absolute method using a fiber optic spectrometer (QE65 Pro, Ocean Optics) combined with an integrating sphere. The mechanical disturbance tests on the PICOF sensor were performed with a maximum bending radius of 2 cm, a maximum stretching strain of 20%, and a maximum swinging frequency of approximately 2 Hz.

**Figure S1.** XRD patterns of LuVO_4_:10%Yb^3+^/x%Er^3+^ (x = 1, 2, 3, 4, 5) and LuVO_4_: y%Yb^3+^/3%Er^3+^ (y = 5, 10, 15, 20, 25, 30, 35) at room temperature.

**Figure S2.** a) UC emission spectra of LuVO_4_: 10%Yb^3+^/x%Er^3+^ (x = 1, 2, 3, 4, 5) under 980 nm Laser excitation at room temperature. b) UC emission spectra of LuVO_4_: y%Yb^3+^/3%Er^3+^ (y = 5, 10, 15, 20, 25, 30, 35) under 980 nm Laser excitation at room temperature. c) Integrated UC emission intensity of LuVO_4_:10%Yb^3+^/x%Er^3+^ (x = 1, 2, 3, 4, 5) and LuVO_4_:y%Yb^3+^/3%Er^3+^ (y = 5, 10, 15, 20, 25, 30, 35) in the range of 510-570 nm.

**Figure S3.** Rietveld refined XRD patterns of LuVO_4_:25%Yb^3+^/3%Er^3+^ sample at room temperature.

**Figure S4.** Thermal imaging photographs of LuVO_4_ powder under 980 nm laser irradiation at different powers.

**Figure S5.** Thermal imaging photographs of LuVO_4_: 3%Er^3+^ phosphor under 980 nm laser irradiation at different powers.

**Figure S6.** Thermal imaging photographs of LuVO_4_: 25%Yb^3+^ phosphor under 980 nm laser irradiation at different powers.

**Figure S7.** Thermal imaging photographs of LuVO_4_: 25%Yb^3+^/3%Er^3+^ phosphor under 980 nm laser irradiation at different powers.

**Figure S8.** Experimental results of UCQY measurements of LuVO_4_: 25%Yb^3+^/3%Er^3+^ under 980 nm excitation.

**Figure S9.** Schematic diagram of the temperature sensing performance evaluating platform.

**Figure S10.** Continuously monitoring results of the input excitation power in LVOYE-composited core-cladding optical fiber at 30 °C.

**Figure S11.** Temperature step change response of the PICOF sensor when immersed into warm water from air at room temperature. The dashed lines denote the air (blue line) and warm water (red line) temperatures measured by a commercial thermocouple.

**Figure S12.** Temperature-dependent absolute sensitivity (*S*_A_) and relative sensitivity (*S*_R_) of the LVOYE-composite PICOF sensor. The values of *S*_A_ and *S*_R_ were calculated by $\text{S}_{\text{A}}\text{ = |}\frac{\text{d}\text{FIR}}{\text{d}\text{T}}\text{|}$ and $\text{S}_{\text{R}}\text{ = }\frac{\text{1}}{\text{FIR}}\text{|}\frac{\text{d}\text{FIR}}{\text{d}\text{T}}\text{| × 100\%}$, respectively.

**Figure S13.** Temperature sensing performances of the LVOYE-composite DCOF sensor. a) Temperature-dependent emission spectra of the DCOF sensor under 980 nm excitation. b) Linear fitting plot of ln(*I*_525_/*I*_554_) versus 1/T. *I*_525_ and *I*_554_ were calculated based on the integrated intensity of emission peaks centered at 525 nm and 554 nm, respectively. c) Actual temperature resolution of the DCOF sensor. d) The continuous monitoring results and measurement deviation from the DCOF sensor at 30 °C. e) Distribution and Gaussian fitting results of the measured temperature at 30 °C from the DCOF sensor.

**Figure S14.** Temperature sensing performances of the LVOYE-composite SCOF sensor. a) Temperature-dependent emission spectra of the SCOF sensor under 980 nm excitation. b) Linear fitting plot of ln(*I*_525_/*I*_554_) versus 1/T. *I*_525_ and *I*_554_ were calculated based on the integrated intensity of emission peaks centered at 525 nm and 554 nm, respectively. c) Actual temperature resolution of the SCOF sensor. d) The continuous monitoring results and measurement deviation from the SCOF sensor at 30 °C. e) Distribution and Gaussian fitting results of the measured temperature at 30 °C from the SCOF sensor.

**Figure S15.** Schematic diagram of a sodium-ion pouch-type battery and a temperature detecting system based on the PICOF sensor and the SCOF sensor.

**Figure S16.** Swelling ratio test results of PDMS film (a), OE film (b), and PICOF (c) in the electrolyte. The swelling ratios were calculated from the mass change before and after immersion, and the reported values are the averages obtained from three independently prepared samples. The dimensions of both the PDMS and OE films are 10 × 10 × 1 mm.

**Figure S17.** Voltage profile of the battery and temperature readings from the PICOF sensor during long charge/discharge cycles at 1 C rate.

**Figure S18.** Photograph of the flexible PICOF sensor implanted in a human-ear model under substantial deformation.

**Figure S19.** Cross-sectional microscope images of the PICOF before (a) and after (b) 100 bending cycles with a bending radius of 5 mm.
